# The Relationship Between Negative Focused Disposition and Suicidal Ideation Among College Students: The Mediating Effects of Somatic Anxiety, General Distress, and Depression

**DOI:** 10.3389/fpsyt.2022.928666

**Published:** 2022-06-28

**Authors:** Guoxiao Sun, Zongyu Liu, Zhiyao Ma, Bob Lew, Cunxian Jia

**Affiliations:** ^1^School of Physical Education, Shandong University, Jinan, China; ^2^Griffith University, School of Applied Psychology, Australian Inst Suicide Res & Prevent, Brisbane, QLD, Australia; ^3^School of Public Health, Cheeloo College of Medicine, Shandong University, Jinan, China

**Keywords:** negative focused disposition, suicidal ideation, somatic anxiety, general distress, depression

## Abstract

**Objective:**

Suicide among college students is a major public health problem. Research has confirmed that negative focused disposition had a significant effect on suicidal ideation. This study aims to evaluate somatic anxiety, general distress and depression as mediators of the relationship between negative focused disposition and suicidal ideation.

**Methods:**

A total of 1798 college students (942 males) were recruited to complete measures of negative focused disposition, somatic anxiety, general distress, positive affect and suicidal ideation. The mediation models were conducted to assess the mediating effects of somatic anxiety, general distress and depression.

**Results:**

There was a significant and positive correlation between negative focused disposition and suicidal ideation in Chinese college students. On mediation analysis, somatic anxiety, general distress and depression mediated the relationship between negative focused disposition and suicidal ideation.

**Conclusions:**

Negative focused disposition appears to directedly associate with suicidal ideation and can indirectly relate to suicidal ideation through the relation with somatic anxiety, general distress and depression. College students with few positive expectations of the future may benefit from interventions focusing on somatic anxiety, general distress and depression.

## Introduction

Suicide among college students is a major public health problem (1, 2), which can take a huge toll on society and cause untold psychological pain to their relatives (3, 4). Individuals with suicidal ideation are more likely to make a suicide attempt (5, 6). Therefore, a better understanding of mechanisms underlying suicidal ideation is the key to reducing suicide among college students (7).

One widely assessed cognitive risk factor for suicide is negative focused disposition, which mainly refers to feelings of worry and pessimism, cognitive rigidity, life dissatisfaction (8). It is also a negative view of the world, including a negative view of oneself and the future (9, 10). Scholars have concluded that people who completed suicide may struggle with negative thoughts until the last moment of their lives (11), which indicates that pessimistic predictions of future events are associated with the risk of suicidal ideation (12). College students are probably under more pressure today than they have been in more than 20 years (13). They may overthink over and even feel pessimistic or hopeless about the future when they meet with difficulties (14). Individuals with perfectionism personality are more likely to cause their compulsive thinking and self-blame for past negative life events, which will aggravate the individual's pain experience, lead to negative psychological states such as despair, and then produce suicidal ideation and serious suicide behavior (15, 16). The Future Disposition Inventory was developed to measure negative focused disposition. This inventory was designed in response to the need for an empirically-validated measure of hopelessness (future disposition) that is associated with suicidal thoughts and behavior (8). Hopelessness is thought to be an independent cause of suicide, according to hopelessness theory, proposed by Beck and his colleagues (17). The theory holds that hopelessness is an important risk factor, which is said to activate the cognitive system, as well as other systems in which suicidal thoughts and behaviors develop. These facts demonstrate a positive correlation between negative focused disposition and suicidal ideation.

Considering the seriousness of suicide among college students, a better understanding of the causes of suicidal ideation in college students with negative focused disposition is urgently needed. However, the mechanism by which negative focused disposition affects suicidal ideation is unclear. The tripartite model of anxiety and depression posits a shared general factor and unique aspects to both anxiety and depression, which was developed to explain both the strong comorbidity and the distinction between them (18, 19). The tripartite model has been widely applicated in adult samples and recently been examined in the youth (20). Understanding the role of dimensions of the tripartite model of anxiety and depression is integral to the evaluation of negative focused disposition as a predictor of suicide ideation. Somatic anxiety, general distress and positive affect are three dimensions of this model. Somatic anxiety is characterized by elevated levels of physiological tension and arousal, depression is lowered levels of positive affect, while general distress shares a number of common features of anxiety and depression (20). Studies have demonstrated that suicidal ideation is positively associated with somatic anxiety (21) and general distress (22), and is negatively associated with positive affect (14).

Somatic anxiety produces obvious physical symptoms such as dizziness, tremors, numbness, or tingling (23). Strohmeier et al. (24) found that feelings of hopelessness about the future are positively correlated with anxiety. Bentz et al. (25) concluded that individuals with a high degree of pessimism about future events had a higher degree of anxiety. The evidence demonstrated that negative focused disposition is positively related to anxiety. Asselmann et al. (26) found that somatic anxiety was one of the main reasons for the occurrence and aggravation of suicidal ideation, which was closely related to suicide. Anxiety was longitudinally associated with suicidal ideation (27), and individuals are more likely to have suicidal ideation when they are anxious, and suicide is often thought of as a means to overcome anxiety or pain in their lives (28). This provides strong evidence to explain the positive relationship between anxiety and ideation. These studies suggested that the influence of negative focused disposition on deliberate ideation may be mediated by somatic anxiety. College students who have a disposition to focus on negative things are more likely to feel worried and feared about the future and eventually may generate the thoughts of killing themselves.

General distress is a non-specific construct including symptoms of psychiatric/psychological disorders, e.g., sadness, anxiety (29). Studies have found that people who see things pessimistically are more likely to experience psychological distress (30) and high levels of hopelessness about the future are associated with high levels of psychological distress (31). Research has shown that when general distress occurred, psychological barriers happened, and suicidal ideation was then developed (32). Those who reported higher levels of psychological distress were more likely to report suicidal ideation than those who reported lower levels of psychological distress (33). Among those with any suicidal thoughts, 71 percent were found to have the highest distress severity (34). Suicidal ideation is a warning sign and symptom of psychological distress (35). Increased psychological distress may also lead students with suicidal ideation to avoid seeking help from family, school or doctors, which is not conducive to improving their suicidal thoughts, emotional and physical problems (32). Considering this, distress may have an indirect influence on the relationship between negative focused disposition and suicidal ideation. College students who have a negative thought about the future may frequently have a feeling of pain and distress and eventually want to end their lives.

Considering that low-level positive affect is indicative of high-level depression (36), our study uses the positive affect dimension in the tripartite model to measure depression. Depression is a common mental disorder that causes psychological distress and can have a significant impact on one's private and public life (37). A person's negative thoughts and feelings about their future is associated with many other mental health conditions, including depression (38). Ballard et al. (9) revealed a significant positive correlation between negative focused disposition and suicidal ideation in undergraduates with moderate to severe depression, while the reverse of the relationships was not verified. Besides, another study has shown that there is a significant positive correlation between depression and suicidal ideation (39). Studies have supported the mediating role of depressive symptoms in the relationship between hopelessness and suicidal ideation (40). Research has confirmed that depression and suicidal ideation are linked to a certain extent (41, 42), which indicates that negative focused disposition can further lead to suicide by producing or even aggravating depression. College students who focus on negative events are tend to have decreased positive affect and aggravated depression and eventually generate suicidal thought.

The above evidence leads us to speculate that negative focused disposition may influence suicidal ideation through anxiety, distress and depression. As far as we know, there are few studies to test the mediating effects of somatic anxiety, general distress and depression between negative focused disposition and suicidal ideation (9). Such a relationship would suggest three potential mechanisms for explaining suicidal ideation and, if replicated, may help to develop specific therapeutic goals for suicide interventions.

Given the strong relationship between somatic anxiety, general distress, and depression with negative focused deposition and suicidal ideation, the current study aimed to investigate a potential model of these factors. Specifically, the purpose of this study was to explore the mediating roles of somatic anxiety, general distress, and depression between negative focused disposition and suicidal ideation in college students to clarify how negative focused disposition affected suicidal ideation. Based on previous studies, we propose the following hypotheses: negative focused disposition can significantly and directly relate to suicidal ideation (H1), negative focused disposition can be associated with the level of suicidal ideation of college students through the mediation of somatic anxiety (H2), general distress (H3), and depression (H4).

## Methods

### Participants and Data Collection Procedure

A total of 1,854 college students were recruited. Data were collected at five campuses (Hongjialou campuses, Xinglongshan campus, Baotuquan campus, Ruanjianyuan campus, Zhongxin campus) of Shandong University in Jinan, Shandong Province. Before our research, researchers printed out the electric questionnaire link and distributed it in libraries, classrooms and dining halls. The trained researchers collected data on basic sociodemographic information, negative focused disposition, somatic anxiety, general distress, positive affect, and suicidal ideation voluntarily. This study was approved by the Ethics Committee of School of Public health, Shandong University (No. 20190912).

Among 1854 self-reported questionnaires that were handed out, 56 pieces of data were eliminated because of the missing items, and the number of data that remained were 1798. The mean age of the participants was 19.931 (SD = 1.684) years. The other socio-demographic information of the participants were listed in Table 1.

**Table 1:** Socio-demographic information of the participants (*n* = 1798).

**Socio-demographic** **variables**	**Categories**	***n***	**Percentage (%)**
Gender	Male	942	46.74
	Female	856	53.26
Residence	Urban	927	51.6
	Rural	871	48.4
Only child or not	Yes	912	50.9
	No	886	49.3
Ethnicity	Han	1696	94.3
	Minority races	102	5.7
Grade	Freshmen	595	33.1
	Sophomores	501	27.9
	Juniors	459	25.5
	Seniors	243	13.5
Father education	Primary school or below	183	10.2
	Junior middle school	566	31.5
	High school	396	22.0
	Junior college	297	16.5
	College	308	17.1
	Postgraduate	48	2.7
Mother education	Primary school or below	344	19.1
	Junior middle school	531	29.5
	High school	321	17.9
	Junior college	307	17.1
	College	256	14.2
	Postgraduate	39	2.2
Marital status of parents	Very harmonious	612	34.0
	Harmonious	551	30.6
	Sometimes make contradictions	444	24.7
	Often make contradictions	81	4.5
	Separated	14	0.8
	Divorced	62	3.4
	One has passed away	34	1.9

### Measures

#### Covariates

The present study considered age, gender, grade, ethnicity, residence, only child or not, ethnicity, education level of father, education level of mother and marital status of parents as covariates, which aimed to ensure the accuracy of data analysis.

#### Negative Focused Disposition

The Negative Focused Disposition was measured by the Future Disposition Inventory (FDI) that was designed in response to measuring hopelessness (i.e., future disposition) which is associated with suicidal thoughts and behaviors (8). The scale is a self-report instrument consisting of 8 items (For example, “I worry that things will never go well for me no matter what I do”) rated on a 5-point Likert scale. The reliability and validity of the instrument have been shown by previous studies (8). The Chinese version also has been supported the strong reliability and validity for Chinese students that come from convenient large public medical universities (43). In this study, the Cronbach's α coefficient of this scale was 0.828.

#### Somatic Anxiety, Positive Affect, and General Distress

The Somatic Anxiety, Positive Affect, and General Distress were assessed using the Anxiety Depression Distress Inventory-27 (ADDI-27) which is a short version of the Mood and Anxiety Symptom Questionnaire-90 (MASQ-90) (44). Some questions such as “Worried a lot about things”, “Felt sad”, and “Felt really happy” were asked to measure somatic anxiety, general distress and depression. All three dimensions are self-report instruments consisting of 9 items rated on a 5-point Likert scale. The reliability and validity of the instrument have been shown by previous studies (44). The reliability and validity of the Chinese version also has been supported in Chinese medical student samples (45). In this study, the Cronbach's α coefficient of somatic anxiety, general distress and positive affect were 0.925, 0.911, and 0.910 respectively.

#### Suicidal Ideation

Suicidal ideation was assessed using the Multidimensional Suicide Inventory-28 (46). In this study, one of the dimensions of the scale, suicidal ideation was used. The suicidal ideation consists of 7 items rated on a 5-point Likert scale. A typical item of the scale is “I have had frequent thoughts of killing myself over the past 2–3 weeks.” The reliability and validity of the instrument have been shown by previous studies in a military trainee sample (46). In this study, the Cronbach's α coefficients of suicidal ideation was 0.928.

### Quality Control

Before the survey, the authors read a lot of relevant literature, discussed with the members of the research group to determine the content of the questionnaire and the scientific and feasibility of its implementation. Five undergraduate students were unified trained to be qualified investigators. When conducting the survey, the investigators uniformly guided the participants to fill in the questionnaire. The purpose of the survey was presented in the questionnaire for the participants to read. The informed consent was obtained before the survey and participants were granted the right to agree or not agree to participate before the survey. After the survey, the data were entered by the uniformly trained data entry researcher, and then the data were logically checked and corrected by two researchers.

### Statistical Analysis

The original data were exported from the Wenjuanxing questionnaire platform (https://www.wjx.cn/). All of the statistical analyses were performed with SPSS version 24.0 (IBM, Armonk, NY, United States) for windows. According to the research purpose, measurement data were expressed as the mean ± standard deviation (SD). A bivariate Pearson correlation was performed to test the level of association among negative focused disposition, somatic anxiety, general distress, depression and suicidal ideation. Moreover, a mediation model to test the direct and indirect effects on suicidal ideation was performed. As in previous research (i.e., (47, 48)], Model 4 in Hayes' PROCESS macro (49) in SPSS (version 3.3) was performed to examine hypothesized relationships by using the negative focused disposition as independent variables, suicide ideation as the dependent variable, and somatic anxiety, general distress, positive affect as parallel mediating variables. The socio-demographic variables were controlled as covariates. The bootstrap method (sampling was repeated 5,000 times) was used to estimate 95% confidence intervals (CIs) (the direct or indirect effect was considered significant if the CI did not include zero) for significance testing of mediating effects. All variables were standardized before entering the mediation model.

## Results

### Common Method Bias Testing

Common variance analysis was applied to the five questionnaires through factor analysis. The chi-square statistic of Bartlett's test of sphericity was significant (KMO = 0.966, *p* < 0.001). After principal component analysis, 5 eigenvalues greater than 1 were extracted. The first factor to explain the variance was 34.857%, which was less than the 40% required by critical standards, demonstrating that the questionnaires used in the current study had no significant issue with common method biases (50).

### Correlation Analysis

Means, standard deviations, and correlation matrix for negative focused disposition, somatic anxiety, general distress, positive affect, and suicidal ideation are presented in Table 2. A bivariate correlation analysis showed that positive affect was significantly and negatively correlated with suicidal ideation; negative focused disposition, somatic anxiety, and general distress were significantly and positively correlated with suicidal ideation (*p* < 0.001).

**Table 2:** Descriptive statistics and correlation matrix for negative focused disposition, somatic anxiety, general distress, positive affect, and suicidal ideation.

**Variables**	**1**	**2**	**3**	**4**	**5**
1 Negative focused disposition	1.000	−	−	-	-
2 Somatic anxiety	0.469^***^	1.000	−	-	-
3 General distress	0.602^***^	0.748^***^	1.000	-	-
4 Positive affect	−0.259^***^	−0.136^***^	−0.263^***^	1.000	-
5 Suicidal ideation	0.434^***^	0.654^***^	0.574^***^	−0.180^***^	1.000
Mean	2.276	1.881	2.209	3.219	1.553
SD	0.765	0.847	0.844	0.856	0.832

*** *p < 0.001. Demographic variables as covariances*.

### Mediation Analysis

Table 3 demonstrated the regression coefficients between negative focused disposition, somatic anxiety, general distress, positive affect and suicidal ideation.

**Table 3:** Regression coefficients between negative focused disposition, positive affect, somatic anxiety, general distress and suicidal ideation.

**Outcome variables**	**Predictors**	**Goodness-of-fit indices**			**Regression coefficients**	
		***R***	***R*** **^2^**	***F***	**β**	***t***
Suicidal ideation		0.482	0.232	23.318^***^		
	Negative focused disposition				0.434	20.272^***^
Somatic anxiety		0.509	0.259	27.028^***^		
	Negative focused disposition				0.470	22.389^***^
General distress		0.626	0.392	49.780^***^		
	Negative focused disposition				0.604	31.767^***^
Positive affect		0.313	0.098	8.356^***^		
	Negative focused disposition				−0.262	−11.305^***^
Suicidal ideation		0.697	0.486	64.412^***^		
	Negative focused disposition				0.114	5.166^***^
	Somatic anxiety				0.512	19.322^***^
	General distress				0.108	3.648^***^
	Positive affect				−0.051	−2.825^**^

** *p < 0.01;*

*** *p < 0.001. Demographic variables as covariances*.

**Figure 1:**
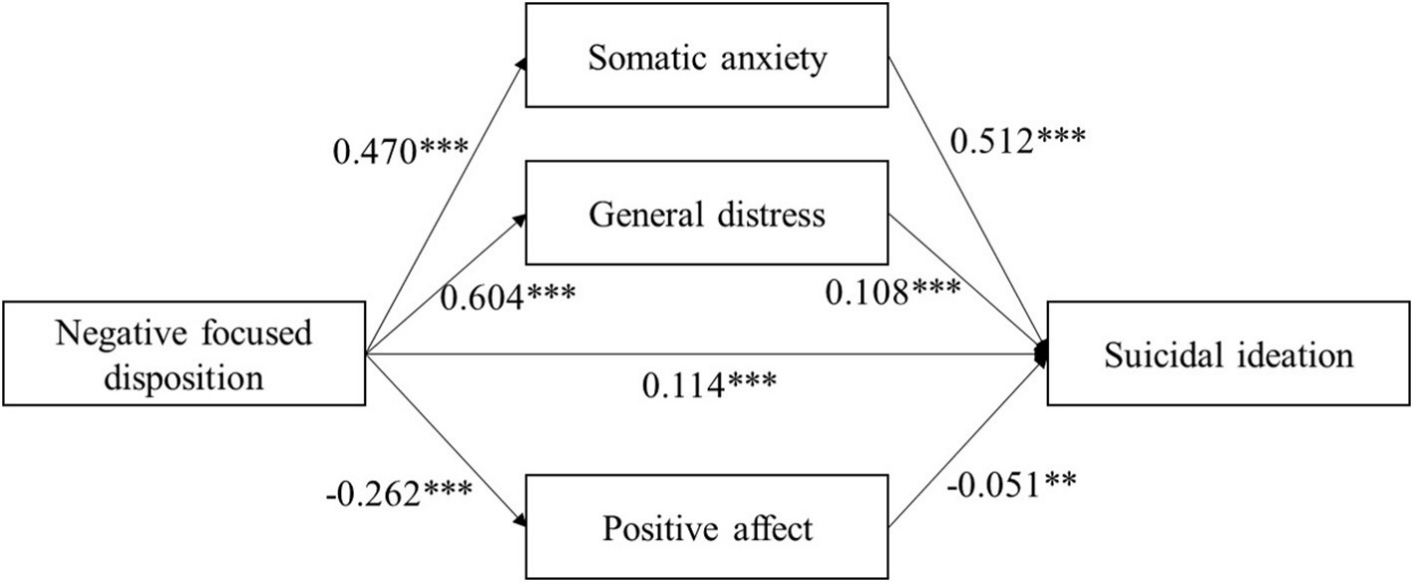
Mediation of somatic anxiety, general distress, positive affect and between negative focused disposition and suicidal ideation. ** *p* < 0.01; *** *p* < 0.001.

Mediation analysis based on 5,000 bootstrap samples was conducted to estimate the indirect effects of negative focused disposition on suicidal ideation mediated by somatic anxiety, general distress, and positive affect. Table 4 illustrates the results of the multiple mediating effects of somatic anxiety, general distress, and positive affect between negative focused disposition and suicidal ideation. The direct effect of negative focused disposition on suicidal ideation was significant (95% CI = 0.071–0.158), and the total indirect effect was also significant (95% CI = 0.276–0.364). The indirect effect of negative focused disposition on suicidal ideation via the mediation of somatic anxiety (95% CI = 0.204–0.278), general distress (95% CI = 0.021–0.111), and positive affect (95% CI = 0.004–0.024) were all significant (Figure 1, Table 4). Besides, significant differences were found between all three indirect effects. The indirect effect of somatic anxiety was the largest (r = 0.241), then general distress (r = 0.065), and positive affect (r = 0.013) was the smallest. The differences of indirect effects between somatic anxiety and general distress, between somatic anxiety and positive affect, and between general distress and positive affect were 0.176 (95% CI: 0.108–0.243), 0.227 (95% CI: 0.188–0.268),0.052 (95% CI: 0.003–0.103) respectively. The CIs did not include zero, indicating that the mediating effect of somatic anxiety was significantly greater than that of general distress and positive affect. The results revealed that there were multiple mediating effects of somatic anxiety, general distress and positive affect between negative focused disposition and suicidal ideation. What's more, somatic anxiety had the greatest mediating effect between negative focused disposition and suicidal ideation.

**Table 4:** Mediating effects of somatic anxiety, general distress and positive affect between negative focused disposition and suicidal ideation.

**Effect types**	**Path**	**95% CI**	**Effects**
Direct effect	Negative focused disposition → Suicidal ideation	0.071–0.158	0.114
Indirect effect	Negative focused disposition → Somatic anxiety → Suicidal ideation	0.204–0.278	0.241
	Negative focused dispositions → General distress → Suicidal ideation	0.021–0.111	0.065
	Negative focused disposition → Positive affect → Suicidal ideation	0.004–0.024	0.013
C1	-	0.108–0.243	0.176
C2	-	0.188–0.268	0.227
C3	-	0.003–0.103	0.052
Total indirect effect	-	0.276–0.364	0.319
Total effect	-	0.392–0.475	0.434

*C1, Somatic anxiety minus General distress; C2, Somatic anxiety minus Positive affect; C3, General distress minus Positive affect*.

## Discussion

The current investigation examined the cross-sectional links between negative focused disposition and suicidal ideation in Chinese college students and investigated the mediating effect of their relationship through somatic anxiety, general distress and depression. In the current study, we found that negative focused disposition was associated with suicidal ideation via somatic anxiety, general distress and depression among a sample of Chinese college students. These findings suggest potential mechanisms through which negative focused disposition can lead to suicidal ideation, and facilitate the development of targeted interventions for individuals to reduce suicide risk in college students.

There are a few studies supporting the positive future thinking - suicidal tendency relationship, but the negative thinking - suicidal tendency relationship is less discussed (51). One important purpose of this study is to provide evidence for the relationship between negative focused disposition and suicidal ideation. Our study showed that negative focused disposition was positively associated with suicidal ideation among Chinese college students, which was consistent with previous studies (9, 12). Hypothesis 1 was supported. Our results also appeared in line with the hopelessness theory (17). According to this theory, individuals with suicidal ideation have increased negative expectations of the future, which can predict future suicidal ideation. Individuals who feel hopeless about the future tend to think that the future will always be negative, cling to a fatalistic view and believe that their situation will never get better (52, 53). Combining the above and our results, we can infer that the more negative thoughts an individual has, the more negative he or she is about the future, the higher the likelihood of developing suicidal ideation.

Although this study implied that negative focused disposition can relate to suicide ideation, considering the overall situation and the internal mechanism of suicide was still vital. Therefore, it is not enough to clearly define the relationship between negative focused disposition and suicidal ideation (54). Our mediation analyses revealed a significant mediating effect of somatic anxiety on the association between negative focused disposition and suicidal ideation among Chinese college students. Hypothesis 2 was supported. This is consistent with previous research, which revealed that negative focused disposition is positively related to anxiety (24). Students who feel pessimistic about the future may feel pressured to escape and subsequently feel anxious. College students face unique stressors of competing responsibilities, managing relationships and personal finances, and having post-graduation employment, which can affect their psychological state [such as feeling hopeless or anxious, (55)]. The anxiety accumulated over a long period may make them feel more and more inextricable and produce suicidal ideation, which may finally turn into suicidal behavior (56, 57). This is consistent with previous research, which found that individuals who are under long-term stress and anxiety are more likely to suicide (58). When the anxiety of college students cannot be effectively addressed, students will attempt suicide (59).

Besides, we found that general distress played a mediating effect in the relationship between negative focused disposition and suicidal ideation, which was also consistent with previous studies (32, 60). Hypothesis 3 was supported. If college students feel negative about the future, they may ruminate on their current plight and feel physical and mental pain as a result (30). Some college students who are under the pressure of homework, projects, and internships may have negative thoughts about doing these tasks better. Faced with constant pressure, they seldom have time to relax after class, which may make them feel distressed (61). It is generally accepted that high levels of negative thinking enhance distress in the face of life stress (62). When the distress reaches a certain level, they may choose suicide as a means of relief.

Moreover, we also found that depression was not only associated with suicidal ideation but also mediated the relationship between negative focused disposition and suicidal ideation. Hypothesis 4 was supported. When an individual has a negative view of the future or unpleasant experiences, it is accompanied by depression (9, 10, 63). Depression is one of the strongest psychological disorders associated with suicidal behavior that will increase the risk of suicide (64). The differential activation hypothesis suggests that depressed emotions are associated with negative and dysfunctional beliefs, which tend to activate suicidal ideation (65). This finding is also supported by Beck's negative depression triad (66), which assumes that negative thoughts about the future are related to negative thoughts about oneself. Besides, the schematic appraisal model of suicide believed that negative information processing biases can affect future evaluation, in which an individual may think that suicide is a way to escape from unbearable emotions or states resulting in developing suicidal ideation (67).

In the present study, we also found that the mediating effect of somatic anxiety on the relationship between negative focused disposition and suicidal ideation was significantly greater than that of general distress and depression. The explanation may be that compared with general distress and depression, negative focused disposition is more likely to make college students feel that their psychological needs are blocked or unable to be realized, which may lead to a sense of anxiety. Their related emotions cannot be adequately communicated and timely mental health support is difficult to obtain. As a result, their longing for life and ideation becomes less strong and may lead to suicidal ideation (21, 68). In other words, college students who felt pessimistic about the future were easily to experience somatic anxiety, which is more difficult to relieve and may ultimately leads to suicidal ideation. Therefore, our results indicate that it is important to consider the role of reducing somatic anxiety in the relationship between negative focused disposition and suicidal ideation.

Theoretically, such mediation models support hopelessness theory (17), which suggests that people who take a pessimistic view of the world become increasingly anxious or distressed by the possibility of negative future events, which increase their likelihood of suicidal ideation. Beck's Negative Depression Triad indicates that depression is the result of feeling negative about the future as well as a key factor in the causation of suicidal ideation (66). Students endorsing greater levels of negative focused disposition will likely respond to cognitive therapy focused on reducing negative attribution. Practically, identification of negative focused disposition and intervention with their positive affect, general distress and somatic anxiety allows for appropriate resource allocation to meet their needs and may reduce the likelihood of progression to suicidal ideation. Government or educators can guide and help students decrease their negative focused disposition (e.g., design specialized online courses and activities or conduct timely psychological counseling), in the true sense to encourage and create conditions for college students to reduce somatic anxiety, general distress and depression, thus reducing the possibility of their suicidal ideation. Positive individualized wellbeing interventions for pessimistic college students to reduce their physical anxiety, depression and general pain levels may help them reduce suicidal ideation and improve their mental health.

Despite these findings, the current research is not without limitations. The most important limitation of these analyses is the use of cross-sectional samples; real mediation requires longitudinal data to determine time priorities. As a result, these results do not provide information on how future personality, depression, and suicidal ideation develop over time, or how these individuals respond to treatment. Secondly, self-reported data have a possibility for reporting bias. Thirdly, as the study was carried out with a sample of college students, the applicability of the research results in different populations (such as teenagers or the elderly), should be verified in future studies. Finally, suicide might be a complex social problem in Chinese culture setting where multiple culture interacts to influence individuals' thoughts and behaviors [see the cultural additivity phenomenon, (69)]. It is suggested that various socio-cultural factors should be taken into consideration to enrich the suicidal ideation model. Additionally, cross-cultural research is expected to examine whether the relationships found may vary by social and cultural context.

In conclusion, there is a significant correlation between negative focused disposition and suicidal ideation; meanwhile, somatic anxiety, general distress and depression play a joint mediating role in the relationship between them. The life of college students is often accompanied by unique environmental and cultural pressures. Future research needs to focus on identifying important and persistent factors that may lead to suicidal ideation and exploring the underlying mechanisms and potential interventions.

## Data Availability Statement

The raw data supporting the conclusions of this article will be made available by the authors, without undue reservation.

## Ethics Statement

Ethics approval was obtained from the Ethics Committee of the School of Public Health, Shandong University (No. 20190912). Participants received an explanation of the study, and they read the instructions in the first part of the questionnaire (which stated that their participation is voluntary and anonymous). Informed consent was obtained from all participants before the start of the survey. Consent was approved by the Ethics Committee. Written informed consent to participate in this study was provided by the participants' legal guardian/next of kin.

## Author Contributions

GS, BL, and CJ designed and implemented this study after obtaining IRB approval, collected data, sorted out and analyzed the data, and wrote the introduction and methods section with post-graduate students ZL and ZM. ZL and ZM participated in the statistical analysis phase of the project, wrote the results part of the paper, and provided detailed feedback on other parts of the paper. All authors approved the final clause.

## Funding

This study was supported by the Social Science Fund of Shandong Province, China (No. 18DTYJ02), the Teaching Research Project of Shandong University (No. XYJG2020025), the Projects of the State General Administration of sports (2022-C-21), and the Young Scholars Program of Shandong University.

## Conflict of Interest

The authors declare that the research was conducted in the absence of any commercial or financial relationships that could be construed as a potential conflict of interest.

## Publisher's Note

All claims expressed in this article are solely those of the authors and do not necessarily represent those of their affiliated organizations, or those of the publisher, the editors and the reviewers. Any product that may be evaluated in this article, or claim that may be made by its manufacturer, is not guaranteed or endorsed by the publisher.
